# “I am not a priority”: ethnic minority experiences of navigating mental health support and the need for culturally sensitive services during and beyond the pandemic

**DOI:** 10.1136/bmjment-2024-301481

**Published:** 2025-04-24

**Authors:** Evgenia Stepanova, Sarah Croke, Ge Yu, Oládayò Bífárìn, Maria Panagioti, Yu Fu

**Affiliations:** 1 Population Health Sciences Institute, University of Newcastle upon Tyne, Newcastle upon Tyne, Tyne and Wear, UK; 2 Division of Population Health, Health Services Research and Primary Care, The University of Manchester, Manchester, UK; 3 Health Services and Population Research, King’s College London Institute of Psychiatry Psychology and Neuroscience, London, UK; 4 Nursing and Advanced Practice, Liverpool John Moores University Faculty of Health, Liverpool, UK; 5 Research and Innovation, Mersey Care NHS Foundation Trust, Liverpool, UK; 6 Primary Care and Mental Health, Institute of Population Health, University of Liverpool, Liverpool, UK

**Keywords:** COVID-19, Depression, Adult psychiatry

## Abstract

**Background:**

Existing health inequalities and the lack of timely and appropriate support have long been a reality for many ethnic minority individuals living with mental health conditions, even before the pandemic. Limited access to services and the absence of culturally or religiously embedded care have led to increased severity of mental health problems.

**Objective:**

To explore the complexity of interactions between ethnic minorities and mental health services and their experiences of seeking and receiving mental health support throughout the pandemic.

**Methods:**

Semi-structured interviews with purposive and snowball sampling of ethnic minorities aged over 18 (n=32) across North East and North West in England were analysed using a framework approach.

**Findings:**

Five themes were generated. Cultural stigma attached to mental health could lead to fear and reluctance to seek support. Individuals struggled to engage with non-culturally sensitive health services. Instead, they indicated a strong preference for wider community support, which continued through the pandemic despite interrupted health services. A collaboration between mental health services and ethnic minority communities was advocated to shape services to cultural contexts and improve patient-centred service delivery.

**Conclusions:**

Ethnic minorities with mental health face significant challenges and disparities in seeking and engaging in mental health services. They often seek support from multicultural community settings even though the support is not specifically targeted at addressing mental health issues. Understanding cultural beliefs, religious influences and family and community structures are necessary components of culturally appropriate care.

**Clinical implications:**

Culturally sensitive mental health services need to be integrated into existing systems through initiating collaborations with ethnic minority communities that tailor services to meet the needs of diverse populations, improving overall engagement and experiences.

**WHAT IS ALREADY KNOWN ON THIS TOPIC**

- Ethnic minorities with mental health conditions face barriers to accessing mental health support.
- There is a cultural stigma attached to mental health in many ethnic minority communities.
- Little research has explored how the experiences of ethnic minorities in interacting with mental health services have evolved during and beyond the pandemic.

**WHAT THIS STUDY ADDS**

- The cycle of hiding away mental health challenges due to stigma and worsened mental health leads to delayed service access in addition to language barriers and inconsistent information on available services.
- Ethnic minorities struggle to engage with health services that are not responsive to meet the health needs of ethnic minorities influenced by cultural contexts.
- A strong preference for community-initiated support which creates a sense of belonging and connection positively impacting their emotional well-being.
- Collaboration between ethnic minority communities and health services is highly encouraged.

**HOW THIS STUDY MIGHT AFFECT RESEARCH, PRACTICE OR POLICY**

- Culturally competent health services are sought to address patients’ diverse needs and provide holistic care.
- Health professionals should cultivate a strong understanding of cultural contexts and integrate cultural competence into their practice.
- Collaboration between health services and ethnic minority communities is to be initiated for improved understanding of context needs and dissemination of accessible services.

## Background

 Lack of timely support has been the reality for many of the ethnic minorities pre-pandemic relating to internalised stigma about mental health services, higher rates of detention, poorer care and reduced long-term recovery rates.^1^,^3^ The pandemic exacerbated existing health inequalities in several ways due to lockdowns, constraints of quarantine and closure of some supporting services. Consequently, ethnic minority groups have disproportionately higher risks of being adversely affected by COVID-19 in the UK, including overall numbers of cases and the relative numbers of critical care admissions and deaths.^4^ They are found to be more prone to having undiagnosed and untreated mental health conditions.^5 6^ Higher levels of anxiety and depression and worse mental health have been reported among ethnic minorities across the pandemic than white people.^7^ For example, Bangladeshi, Indian and Pakistani males experienced the highest average increase in mental distress compared with white British males.^8^

However, evidence indicated that individuals from ethnic minority communities were less likely to access timely mental health support in primary care. Instead, they often entered the healthcare system through acute care pathways or crisis services and were more likely to be diagnosed with severe mental health illnesses compared with the white British population.^9^ Those with pre-existing mental health conditions faced heightened challenges, including isolation, loneliness, domestic abuse and barriers to accessing mental health services, particularly with the transition to remote care.^10^ Although fewer individuals, especially those from disadvantaged backgrounds, sought support from their general practitioner (GP), the caseload in secondary care (primarily urgent and emergency cases) increased.^11^ Ethnic minorities were also 40% more likely than white individuals to access mental health services through the criminal justice system,^9^ suggesting that support may only become available once conditions have deteriorated.^12^ Consequently, existing mental health services have struggled to meet the increased demand effectively.^13^

For individuals who accessed both medical and social support services, the care received was often perceived as inadequate in addressing their needs. The absence of culturally or religiously relevant discussions, along with limited access to information necessary for informed consent, contributes to suboptimal care experiences. These challenges, compounded by cultural stigma and fears of discrimination, further hinder access to mental health services.^14^

Despite increased challenges faced and deepened inequalities, limited research has investigated the experiences of ethnic minorities engaging with mental health services or how these interactions have evolved during and beyond the pandemic. The few existing qualitative studies primarily focus on changes in mental health status^15^ and perinatal mental healthcare^16^ within ethnic minority communities, leaving a gap in understanding their multilayered experiences and holistic journeys during this time. Additionally, there is limited insight into the complex interplay between ethnic minority communities and the evolving mental health services during and beyond the pandemic period.

### Objective

This study aimed to explore the nuanced interactions between ethnic minorities and mental health services by examining their experiences of seeking and receiving support for mental health conditions throughout and beyond the pandemic. By understanding their service engagement and coping strategies, we aimed to capture how these experiences have shaped their mental health and overall well-being. This research forms part of a larger mixed-methods project^17 18^ focused on establishing culturally competent mental health services by integrating electronic health records and qualitative data, thereby contributing to the consensus on core characteristics of effective mental health service provision.

## Methods

### Study design

This study was conducted in the North East, North Cumbria (NENC), and North West of the UK, regions with high mental health needs but limited research activity.^19^ We conducted in-depth, semi-structured interviews using purposive and snowball sampling methods to recruit ethnic minorities with diverse mental health conditions. This dual focus aimed to provide a comprehensive understanding of the challenges faced by different ethnic minority communities. Participants were recruited through local community organisations supporting ethnic minorities, with study information sent to 19 organisations in NENC and 8 in the North West and Merseyside. Recruitment materials were translated into five languages, and follow-up contacts were made as needed. To capture varied experiences, we also reached out to primary and secondary care settings and promoted the study on social media for self-referral.

Data were collected with written and electronic informed consent and securely stored on the University server.

### Study participants

Eligible participants were adults (18+) from ethnic minority backgrounds who had accessed mental health services for conditions diagnosed before March 2019. Individuals in palliative care were excluded.

### Data collection

Interviews were conducted either remotely or in person between March and September 2023 by two experienced qualitative researchers from two different universities. Participants received an information sheet and consent form in advance, with consent reconfirmed and recorded before each interview. The topic guide (box 1), based on evidence from a review of post-pandemic mental health service changes, was co-produced and piloted with a project advisory group of ethnic minority individuals, carers and clinicians, who collaboratively agreed on terminology, key phrases and areas of discussion. Discussions covered experiences with formal and informal support, service engagement, coping strategies across pandemic phases and suggestions for improvements.

Box 1 **Interview topic guide**

**Demographics**

- Participant level demographics including ethnicity, gender, marital status, age, employment.

**Understanding mental health needs and experiences of living with mental health**

- Current mental health well-being.
- Strategies for managing mental health conditions including seeking mental health support.
- Support received for mental health needs.

**Experiences of managing mental health conditions pre-pandemic**

- Details of support/care received for mental health needs.
- Facilitators and barriers to seeking and engaging mental health services.
- Wider support from communities, peers and family.

**Experiences of managing mental health conditions during and beyond the pandemic**

- Details of support/care provided for mental health needs.
- Changes to seeking and engaging mental health services and consequences.
- Experiences of tele-mental healthcare.
- Wider support from communities, peers and family

**Ethnicity and mental health services**

- Key characteristics to define culturally competent care.
- Perception on implementing culturally competent services for mental health needs.

### Data analysis

With informed consent, interviews were recorded, transcribed verbatim and anonymised by assigning participant IDs and removing identifiable information.

Data were analysed using a framework approach^20^ which allowed a systematic way to manage and interpret data through a set of predefined stages: familiarisation with the data, identifying a thematic framework, indexing, charting, mapping and interpreting the findings. A framework matrix was generated for each transcript to help organise data according to key themes and subthemes, and the analytical framework evolved as coding progressed. This enabled us to track patterns within and across cases while ensuring that all aspects of the data were comprehensively explored. The use of the framework approach facilitated transparency in how themes and subthemes were developed and organised. It also allowed us to compare and contrast data across different participant groups (eg, by ethnicity or mental health condition), which helped to reveal patterns that might not have been as apparent in a less structured analysis.

To ensure validity, two researchers independently double-coded a sample of three interviews. The research team and the advisory group collaboratively reviewed emerging analyses, addressing discrepancies and reaching a consensus on the framework. NVivo V.12 was used for data management and analysis.^21^

## Findings

In total, 32 individuals took part and one interview was undertaken with an interpreter. Table 1 shows participant characteristics. Each interview lasted between 25 and 70 min, resulting in five themes. Tables2  3 show study themes and study quotations respectively.

**Table 1:** Participant characteristics (n=32)

Sociodemographic characteristic	Data
Age	
21–30	7
31–40	12
41–50	8
51–60	4
61–70	1
Gender	
Male	16
Female	15
Transgender	1
Ethnicity	
African	10
Bangladeshi	4
Caribbean	7
Chinese	1
Indian	1
Iranian	1
Latin American	2
Pakistani	3
Somali	1
Sri Lankan	1
Mixed	1
English Language proficiency	
As the first language	16
As the second language	15
Required an interpreter	1
Marital status	
Single	16
Married/civic partner	9
Living with partner/engaged	3
Separated	2
Divorced	2
Employment	
Student	2
Paid employment—part-time	10
Paid employment—full-time	3
Self-employment	1
Unemployed—looking for work	5
Unable to work due to illness/disability	10
Retired	1
Mental health status	
Stable	13
Varied	8
Poor	10
Very poor	1

**Table 2:** Study themes and subthemes

Theme	Subtheme
Barriers to managing mental health challenges	Cultural stigma/ignorance attached to mental health
	Lack of understanding and support
	Fear of negative consequences
Limited engagement with health services	Language barrier
	Not seen as a priority
	Staying away from services
	Service was not culturally sensitive
	Avoiding professionals with same ethnic minority backgrounds
Preference for community support	A sense of belonging with the community
	Non-judgemental peer support
Interrupted service but relying on community support throughout the pandemic	COVID-19 worsened mental health and engagement with professionals
	Sustained need for mental health support post-pandemic
	Relying on family and community support
	Desire for continuity of care
Service-community collaboration to be initiated	Training on culturally responsive care
	Holistic care
	Seeking collaboration between mental health services and communities

**Table 3:** Quotations

1	“The kind of mentality that we have in Africa, is those that will not wear clothes at all, and they will be moving around naked.”
2	“Back in [South Asian country] we didn’t know…I never knew anything like mental health. Back over there, if you suffer something like that, she’s gone mad.”
3	“When I explain it [mental illness] to them, they're like, “You seem to get up every day”, but most days I struggle.”
4	“People started criticising me, always believing in devils and things like that. But I didn’t know which way to go. Doctors not understanding me, family’s not understanding me.”
5	“Language barriers or perceptions, that’s one part, but also think about the system, it’s a totally different system, medical system here… we don’t know what type of support we can get from the NHS so that’s an issue.”
6	“You feel like hopeless; you don’t know what you are going to do. I thought myself better to die myself…You don’t know anyone here. So it’s increased the level of depression.”
7	“I would never admit it [post-natal depression]… I felt like if I admit that I don’t feel good, they’re going to take my baby away from me. I just suffered in silence.”
8	“I was having pins and needles in my head, I went to my GP, I didn’t know how to explain it so I just explained it the way I thought people knew and I said, ‘oh I feel like ants walking in my head.’ The first thing that the doctor did was check my head. I was like no, no, no….”
9	“So, she told me I need to look for some therapy or maybe some therapies in my own language.”
10	“And everything was by phone and my English in that point was really bad. And I don't want to use a translator because it’s very personal, it’s very sensitive for me to speak about my thing.”
11	“I called every day for all the week I couldn't find an appointment. I don't want to think this but sometimes the situation made me think, I'm not a priority.”
12	“I met the psychiatrist, we spoke briefly and then I was prescribed some medication and that was really the end of it. I think I had one or two follow-up appointments, but they weren't really interested.”
13	“The sense of rush. When I've been in a virtual session. We always seeming to be in a hurry and quickly get done with the meeting.”
14	“If they come for visit, I would just force myself to sit with them and talk to them, not for long…The only thing in my mind that I want to get away from there.”
15	“You get abuse from the patients. So patient-to-patient abuse. So the patient will call the p-word or the n-word to other patients. The service professional will do nothing about it. Oh, he’s not well, he’s doesn't know what he’s saying. Yeah, but that’s not acceptable. So you're really ill, trying to recuperate, when you're in there you're worried and you don't feel safe too, because you have other patients who are, let’s say, unpredictable with different mental health issues and conditions. And you get exposed to that and you get racism on top of that.”
16	“I said to them [clinicians] that in our culture, I need a purpose, I need to say where I’m going, the GP said to me, “Do you ask your husband when you need to go to the toilet?”
17	“I always felt like I was labelled, I was stigmatised from being in a minority and putting my voice across.”
18	“I've realised that the darker your skin is, the worse it is for you, they [clinicians] don't take me that I've got diagnosed mental health conditions…I'm looked at as an angry black woman. Then the security guard will kick me out of the hospital while I'm having a meltdown.”
19	“I’m quite open…but I avoid talking to doctors from [Far East] background because I feel like I’m not sure. It’s very strange, even they’re professional… I don’t want to talk my issues to any [Far East] doctors.”
20	“Because I was African and he [GP] was African, he was saying basically you're imagining stuff, or something like that.”
21	“[BAMER community centre] got involved with me in the first time I was sectioned, and I’ve been coming here 11 years”
22	“The community centre is more inviting for me personally and the community centre is great and multicultural and sensitive to those sort of things…it makes it a bit easier.”
23	“So at times they bring something for us to practice, like knitting something, like some games and books for us to read… Regular meetings, handcrafts, at times we have in the garden planting some seed, cutting the grass, cooking in the kitchen. All of us, we just sit down and have a meal together and they talk to us, we joke, then everybody will go home.”
24	“Just during the pandemic I also developed health anxiety, you know with stress and anxiety they have different variations.”
25	“during COVID, everything stopped so those two years impacted me in quite a big way.”
26	“It was more rushed, you were distanced… there was that not that closeness that we started off with, so I would say I didn’t get the best benefit from the [online] sessions really.”
27	“I think accessing service became more stressed in the sense that the system was stressed at that point in time. Then it was really challenging and hard to access all care, you know, like the rules before the pandemic.”
28	“For about two years I haven't been able to get a GP appointment. So, I've been on the same medication ever since. I don't think that medication has helped me or has made me stable.”
29	“One wish that if it would be repeated or maybe not all ten sessions but just a recap, it would strengthen one much more and I think also mental health is something that stays with you for years, there are flickers and so on.*”*
30	“I gave up with remote therapies because they just made me feel unwell and if it wasn't for my friends and family at the time it wouldn't have helped me and support, I don't think I would have been alive by now.”
31	“The support I’ve had from community organisations, that has been, I think, the most effective for me, thus far, in keeping me stable and on the path to getting better… There’s opportunities for workshops and things to give you confidence and just to support people with their mental health and things.”
32	“Keep an eye on them, and try and bring them back up every three weeks so you carry on, what’s happening with them.”
33	“It would be repeated or maybe not all ten sessions but just a recap, it would strengthen one much more.”
34	“Cultural training is a massive, massive element. You need cultural training.”
35	“It would be good for the NHS to have a wider awareness, and they probably do, but everywhere, even in rural areas, some sort of raising awareness of multiculturalism or something like that. I don’t know how…being sensitive to different backgrounds. I mean, they might not come across it any time in their life but they might. Maybe that could be quite a good training.”
36	“Stuff consider the different cultural beliefs and ways of life instead of just following procedure that you are doing… maybe your culture doesn’t allow you to talk to men if they are a woman, all of this consideration should be in place.”
37	“…only tablet and medication is not enough. They need to…how to call it, emotional support and like a therapy service. It’s very important.”
38	“I wish there were maybe more instructions or more information for minority groups on what type of support you can get from the NHS and where, and any other further signposting information for them.”

### Barriers to managing mental health challenges

#### Cultural stigma/ignorance attached to mental health

Mental health was widely perceived as a sensitive and taboo subject, rarely acknowledged within participants’ cultural contexts, contributing to significant “stigma and silence”. Mental health struggles were seen as so distant from their cultural understanding, illustrating how mental health was not conceptualised within their communities. This lack of recognition made it difficult for individuals to address their struggles, leading to prolonged suffering in silence (table 3, Quotes 1,2).

#### Lack of understanding and support

Mental health was described as invisible and not understood by others, making it challenging to open up and ask for support (table 3, Quotes 3,4).

Participants indicated limited support, attributing this to the stigma associated with being non-natives, coupled with their language and cultural barriers limiting their management strategies. Consequently, they were unaware of appropriate actions, leading to hesitation or delay in seeking help (table 3, Quotes 5,6).

#### Fear of negative consequences

Some expressed a deep fear, particularly among parents, that disclosing their mental health struggles could be interpreted as an inability to care for their children, potentially triggering interventions from child protection services (table 3, Quote 7).

### Limited engagement with health services

#### Language barrier

Some participants, though fluent in English for daily tasks, struggled to describe symptoms using medical language or English terminology, leading to confusion or misinterpretation by clinicians. Non-native speakers found this even harder, leading to frustration and feeling misunderstood (table 3, Quote 8).

Several interviewees identified language as a barrier, from discovering support options to accessing available therapy, a challenge worsened by limited alternatives (table 3, Quotes 9,10).

#### Not seen as a priority

Most felt their mental health was not treated as a priority, with long waiting lists and prolonged delays in accessing care, leaving them feeling unsupported. The extended waiting periods often exacerbated their conditions, leading to deterioration of their mental health (table 3, Quote 11).

For those participants who received services, many reported that their consultations felt rushed and their symptoms were not thoroughly explored, with only a brief focus on their immediate concerns, while underlying issues were overlooked (table 3, Quotes 12,13).

#### Staying away from services

Some participants expressed fear of engaging with services due to past experiences, such as being sectioned or having children taken away. They reported pretending to be ‘normal’ and avoiding seeking help in order to steer clear of the system (table 3, Quote 14).

Some also reported racism from other patients during their time in inpatient care. This made an already challenging situation significantly more difficult, leading to feelings of mistrust and fear while in treatment (table 3, Quote 15).

#### Service was not culturally sensitive

Many participants felt their cultural, religious and social contexts were inadequately addressed in treatment, reflecting a lack of cultural sensitivity—defined as the failure to recognise and adapt to diverse values, beliefs and practices. This alienation of individuals eroded trust in healthcare providers and reduced their likelihood of seeking future support. The failure to connect with ethnic minority communities hindered culturally responsive care, perpetuating disengagement and exacerbating disparities in mental health service access and utilisation (table 3, Quotes 16,17).

This issue was particularly acute for individuals experiencing severe mental health crises. Participants in these situations felt that the lack of cultural sensitivity was especially harmful during critical moments. Due to the urgency and intensity of their needs, the inability of care professionals to incorporate cultural understanding or personalised care further discouraged them from seeking services (table 3, Quote 18).

#### Avoiding professionals with same ethnic minority backgrounds

Some participants expressed strong concerns about the idea of seeking help from care professionals who shared their ethnic or cultural backgrounds. This hesitation stemmed from the cultural stigma around mental health, creating a unique barrier to accessing care. Many feared that seeking support from a provider with a similar background might risk exposing their mental health struggles within their community, leading to judgement, gossip or potential breaches of confidentiality (table 3, Quotes 19,20).

### Preference for community support

#### A sense of belonging with the community

All participants described connections with local, diverse community groups, some culturally specific and others GP-referred. These settings provided a safe space for sharing and receiving support, often offering guidance on accessing formal mental health services. Although not designed specifically for mental health support, these informal networks were preferred by many, as they fostered a sense of connection and belonging. For many, community support was essential for coping and socialisation (table 3, Quotes 21,22).

#### Non-judgemental peer support

Some participants expressed a preference for engaging with multicultural communities over local ones, where personal connections could lead to judgement or stigma around mental health struggles. These multicultural networks were seen as accessible, non-judgemental and more aligned with participants’ cultural and social identities. Consequently, these relationships were valued as essential for maintaining mental health, especially when formal services were unavailable or challenging to access (table 3, Quote 23).

### Interrupted service but relying on community support throughout the pandemic

#### COVID-19 worsened mental health and engagement with professionals

The pandemic magnified pre-existing challenges rather than being the root cause for accessibility, stigma and long waiting lists. Many experienced worsened mental health due to changes in healthcare delivery, including remote care, service closures, early discharge and inconsistent quality (table 3, Quote 24).

The shift to remote services posed accessibility challenges, especially for those without reliable technology or private spaces, often leaving them feeling disconnected from needed support (table 3, Quotes 25,26).

Many expressed concerns that mental health services would not have returned to pre-pandemic levels, with limited access and long waitlists still causing anxiety and frustration for those seeking help (table 3, Quote 27).

#### Sustained need for mental health support post-pandemic

Though interviewed post-pandemic, many still faced significant barriers to care access (table 3, Quote 28).

Others emphasised the value of ongoing follow-up support, including opportunities to repeat therapy (table 3, Quote 29).

#### Relying on family and community support

Family and community support served as vital resources for coping with mental health challenges when access to formal services was inconsistent before, during and beyond the pandemic. These informal networks offered crucial emotional support, fostering connection and reducing isolation during periods of heightened stress and uncertainty (table 3, Quotes 30,31).

#### Desire for continuity of care

Participants expressed a strong desire for consistent, ongoing care that would enable the development of meaningful therapeutic relationships, where providers understood their history, progress and unique challenges post-pandemic (table 3, Quote 32).

They emphasised that even a brief recap or check-in could be valuable in maintaining their connection with care professionals and ensuring that their mental health progress was on track. For many, these follow-ups served as a way to feel supported and reassured, even if the interaction was short (table 3, Quote 33).

### Service-community collaboration to be initiated

#### Training on culturally responsive care

Some felt that being judged by their appearance led to misunderstandings or misdiagnoses, particularly when their behaviour was influenced by their mental health condition. They expressed a strong preference for being treated as individuals with mental health concerns rather than being categorised or judged based on their appearance or cultural background (table 3, Quotes 34,35).

Training to improve cultural competency was seen as essential to providing more effective, respectful and individualised care. Interpreters were also advised to be trained to understand mental health terminology and nuances (table 3, Quote 36).

#### Holistic care

While medication was often seen as necessary for stabilising symptoms, many participants felt that it was insufficient on its own and highlighted the importance of multi-component care in managing their mental health conditions (table 3, Quote 37).

#### Seeking collaboration between mental health services and communities

Many emphasised the need for collaboration between health services and ethnic minority communities to enable knowledge exchange which would have helped remove the stigma and encouraged taking appropriate actions (table 3, Quote 38).

## Discussion

The study highlighted shared challenges in managing mental health across ethnic minority groups before, during and beyond the pandemic, including stigma and non-culturally sensitive services. These issues, compounded by pandemic restrictions, contributed to varied health outcomes and unmet service needs.

**Figure 1:**
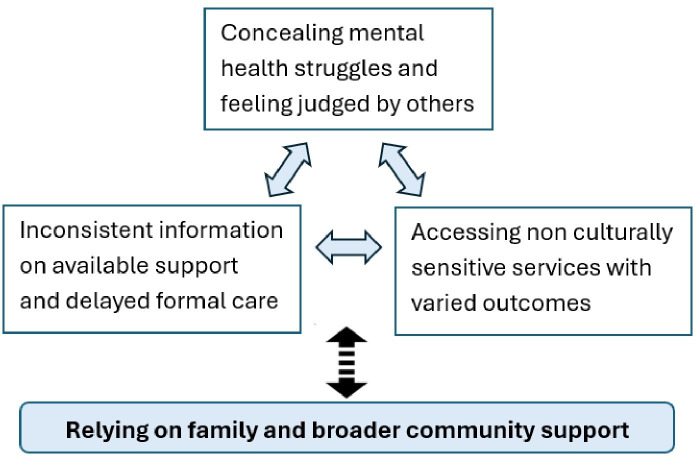
Conceptual model of disconnected mental health services and ethnic minority community support.

A conceptual model shown in figure 1 is developed to illustrate the experience of ethnic minorities living with mental health conditions as we observed them, and their interactions with health services. Our findings extend to previous research exploring mental health in ethnic minority populations,^22^,^24^ demonstrating the conflicts between cultural and religious traditions and the management of mental health. Cultural and religious communities serve as sources of comfort and reassurance, providing members with a sense of continuity and stability in a Western system new to them with cultural shocks and language barriers. However, mental health is not always recognised as a legitimate or accepted concept in many cultural and religious traditions. It has been viewed through alternative lenses for years, for example, spiritual or moral weakness instead of medical or psychological conditions. This perception creates barriers to seeking help due to fear of judgement or ostracisation. Consequently, individuals may avoid formal care, exacerbating mental health issues and potentially leading to severe outcomes such as self-harm. Changes around social norms and practices within these communities, during the pandemic, compounded such challenges.

This study sheds light on the experiences of ethnic minorities accessing and engaging with mental health services before, during and beyond the pandemic. Many people delay seeking care until a crisis occurs, often due to language barriers, cultural stigma and limited awareness of available support. Initial reliance on GPs and family members can lead to inconsistent information and further delays. When services fail to incorporate cultural perspectives, individuals turn to community settings for emotional support, where a sense of belonging positively impacts well-being, even if mental health needs remain unmet.

Building trust is seen as key to engagement, shaped not only by individual provider interactions but also by the broader healthcare system. Where individuals feel their cultural values are acknowledged and integrated into care, they are more likely to engage with services.^25^ However, language barriers and cultural stigma can create a dual challenge for ethnic minority communities, reducing trust and engagement with mental health services and healthcare professionals as well as limiting access to reliable mental health information. The lack of direct translations for medical terms reduces the effectiveness of translation services, while stigma prevents open discussions with community leaders or families. In addition, limited health literacy—especially among disadvantaged groups—exacerbates difficulties in navigating mental health services and communicating symptoms. Over 40% of UK adults struggle to understand general health information, with 60% facing challenges when numbers and statistics are involved, further hindering access.^26^

Culturally appropriate care^27^ extends beyond translation services to encompass an understanding of cultural beliefs, religious influences and family structures in treatment approaches. Evidence suggests that community-based models, peer support and faith-based initiatives enhance engagement and outcomes.^28^ Culturally responsive care should not be the sole responsibility of certain professionals, but must be embedded across the system. Training all clinicians and healthcare staff in cultural competence ensures services are inclusive, accessible and tailored to diverse populations. Collaborations between healthcare providers and ethnic minority communities are essential in designing and delivering culturally sensitive mental health services. Engaging community leaders, incorporating lived experiences and adapting services based on continuous feedback can reduce stigma, improve trust and enhance resource utilisation. A culturally informed approach is necessary to foster equitable mental health support that meets the diverse needs of all individuals.

Although we explored the impact of the pandemic on mental health service engagement, our findings did not reveal challenges for ethnic minority communities that were only specific to this period. Barriers such as long waiting times, accessibility issues and reliance on informal support were all features before the pandemic, and their ongoing nature reflects broader systemic shortcomings rather than pandemic-specific effects. Similar challenges are reported globally among ethnic minority communities,^29 30^ suggesting these issues stem from migration and systemic inequities rather than COVID-19, reinforcing the broader need for culturally responsive and accessible mental health services. Some of the barriers, such as access, remain a key issue post-pandemic, which was repeatedly highlighted by participants.

### Strengths and limitations

The geographical spread, multicultural backgrounds and range of mental health conditions of participants allowed for a wider range of experiences to be investigated. The study explored their interactions with mental health services, from seeking support, service access and admission to inpatient stay. Given that this is an under-represented group in clinical practice and that seeking mental health support is frequently an involuntary and distressing process, it is notable that there was a significant degree of consistency among participants. This suggests that the main issues and challenges have been robustly identified by this study.

Due to our efforts to interview participants from a range of ethnic groups, most were identified in community settings. Although we aimed to capture not only a diverse range of ethnic groups but also individuals with varied mental health conditions, the sample was heavily populated by individuals of African and South Asian heritage, which may limit the generalisability of our findings to other ethnic minority groups. Furthermore, all interviewed had existing mental health conditions pre-pandemic and interviews were conducted after the end of pandemic restrictions. While we used open-ended questions, prompts and follow-up discussions to aid memory recall and elicit detailed responses, this timing might have influenced their ability to recall and reflect in detail on their entire experience of accessing support during this period.

### Clinical implications

Several implications for clinical practice can be drawn from this study. Culturally appropriate care should be a fundamental aspect of all clinical practice. By integrating cultural competence training and practices, clinicians can develop more effective treatment plans and deliver person-centred care that is responsive to the diverse needs, values and preferences of individuals from various backgrounds. Ensuring that all healthcare providers are equipped with the skills and awareness to offer culturally responsive care promotes inclusivity, strengthens patient–provider relationships and improves the overall quality of mental health support.

Our findings also underscore the importance of initiating collaboration between mental health services and ethnic minority communities. This collaboration should focus on educating and disseminating knowledge, as well as providing accessible information to support individuals experiencing mental health conditions. Such a proactive approach can enhance the understanding of cultural needs and preferences, which in turn can guide more effective and tailored use of health resources as well as improved service delivery and better engagement.

## Conclusion

This study provides evidence that ethnic minorities with mental health conditions face significant challenges in seeking and receiving mental health services, which often limits their contact and engagement with current care options. This evidence relates across the period before, during and beyond the pandemic. Understanding cultural beliefs, religious influences and family and community structures are therefore recommended as necessary components of culturally appropriate care.

To address these challenges, healthcare systems should integrate culturally sensitive care, prioritising cultural competence training, community collaboration and tailored service delivery for all clinical practices. Engaging ethnic minority communities, leveraging peer and faith-based support, and embedding cultural and religious understanding into care frameworks are essential steps toward equitable and effective mental health support. By fostering trust and inclusivity, services can better meet the needs of diverse populations, improving engagement and outcomes.

## Data Availability

Data are available upon reasonable request.
